# Heat Shock Protein 104 (*Hsp104*) in the Marine Diatom *Ditylum brightwellii*: Identification and Transcriptional Responses to Environmental Stress

**DOI:** 10.3390/genes16121408

**Published:** 2025-11-26

**Authors:** Han-Sol Kim, Jong-Won Lee, Jang-Seu Ki

**Affiliations:** 1 Department of Life Science, Sangmyung University, Seoul 03016, Republic of Korea; r20240165@smu.ac.kr (H.-S.K.);; 2 Institute of Natural Science, Sangmyung University, Seoul 03016, Republic of Korea

**Keywords:** heat shock protein 104, *Ditylum brightwellii*, diatom, environmental stress, biomarker

## Abstract

Backgrounds: The marine diatom *Ditylum brightwellii* has been widely used as a model species for ecotoxicological assessments in marine environments. Heat shock proteins (Hsps) function as molecular chaperones that protect cells under diverse stress conditions. Of them, Hsp104 participates in the protein restoration system by reversing protein aggregation. Methods: In the present study, we determined the full-length sequence of *DbHsp104* in *D. brightwellii* using transcriptome sequencing and gene cloning. Results: The open reading frame (ORF) was 2745 bp in length, encoding a protein of 915 amino acids (101.15 kDa). Phylogenetic and domain structural analysis revealed that DbHsp104 possesses conserved features of eukaryotic Hsp104. In addition, transcriptional responses of the gene were evaluated after exposures to thermal stress at 20, 25, and 30 °C, and heavy metals and endocrine-disrupting chemicals (EDCs) for 24 h. Relative gene expression analysis showed that *DbHsp104* was significantly up-regulated under thermal stress and copper exposures, peaking at 4.87- and 5.55-fold (*p* < 0.001) increases, respectively. In contrast, no significant changes were observed in response to nickel, bisphenol A (BPA), polychlorinated biphenyl (PCB), and endosulfan (EDS) treatments. Conclusions: These results suggest that *DbHsp104* is specifically responsive to acute stress induced by thermal stress and copper, highlighting its potential as a molecular biomarker in marine environments.

## 1. Introduction

Diatoms are unicellular phytoplankton belonging to the group Bacillariophyta, accounting for approximately 40% of total primary production in marine ecosystems [1,2]. They are abundant in coastal ecosystems and play essential roles in ecological processes, particularly through their involvement in the global biogeochemical cycles of carbon, nitrogen, and silicon [3]. Moreover, their rapid cell growth leads to their widespread global distribution and sensitive reactions to environmental fluctuations [4]. Collectively, these features underscore the ecological significance of diatoms and their adaptability to diverse environmental conditions.

Due to their broad occurrence and ecological significance, diatoms are recognized as important biological indicators for monitoring both short-term and long-term environmental changes [5]. They have become an integral part of laboratory-based toxicity tests, ecological risk assessments, the development of water quality guidelines, and even investigations into the bioavailability and impacts of pollutants [6]. Over the past three decades, diatoms have been extensively employed in aquatic risk assessments, demonstrating their ongoing relevance and utility for environmental monitoring purposes [7,8,9,10]. Changes in their population structure, morphology, or molecular traits can indicate early ecological stress, highlighting their suitability as test organisms in ecotoxicity assessments [11].

When exposed to various chemical contaminants, diatoms show significant changes in an array of physiological, morphological, and molecular responses [12,13,14,15]. In detail, heavy metals and organic pollutants decrease cell growth, chlorophyll, and photosynthetic efficiency and alter antioxidant enzyme activities [16,17,18]. Such ecotoxicological disturbances often modulate stress-responsive genes, such as those encoding antioxidant enzymes, and molecular chaperones such as heat shock proteins (Hsps) [19,20,21,22]. Notably, Hsps are induced by a wide range of environmental stresses such as changes in temperature and salinity, and organic and inorganic pollutants [20,21,22,23]. When cells are damaged, Hsps function as chaperones that promote protein folding, transport, degradation, and stabilization, thus preserving cellular proteostasis [20]. Based on their molecular weight and sequence homology, they are classified into five major families: small Hsps (sHsps), Hsp60, Hsp70, Hsp90, and Hsp100 [24]. Among those, Hsp104 is a member of the Hsp104/ClpB family, which functions as a disaggregase [25,26].

Hsp104/Clp is a hexameric AAA+ ATPase and proteolytic enzyme that has been identified primarily in bacteria, plants, and yeast [27]. In this regard, this enzyme participates in maintaining cellular proteostasis, particularly under stress conditions that promote protein denaturation and aggregation [28,29,30]. Hsp104 has been observed to form asymmetric ring-shaped structures containing multi-domains [31]. In detail, the N-terminal domain (NTD) contains two ATPases associated with various cellular activities (AAA+) and two types of nucleotide-binding domains (NBD), termed NBD1 and NBD2, while the coiled-coil middle domain (MD), which mediates interaction with Hsp70, is inserted at the C-terminal end of NBD1 [32,33,34]. In contrast to ClpB, Hsp104 contains an additional short extension at the C-terminal end, which is utilized for hexamerisation [35].

The Hsp104/ClpB family has been extensively studied in bacteria, plants, and yeast [36,37,38], but research on the gene expression remains limited in diatoms. In the present study, we determined the full-length sequence of Hsp104 from the marine diatom *Ditylum brightwellii* (*DbHsp104*). Also, we characterized its structural motifs and analyzed its phylogenetic relatedness to other forms of Hsp104. In addition, we evaluated the transcriptional responses of *DbHsp104* under diverse stress conditions, including heat stress, and under the influence of diverse heavy metals and endocrine-disrupting chemicals (EDCs). Subsequently, based on the transcriptional responses of *Hsp104* to stress and toxicant exposure, we evaluated the biomarker potential of *DbHsp104*. The test species *D. brightwellii* has been used as a model for ecotoxicology research for the past 40 years [7,39].

## 2. Materials and Methods

### 2.1. Cell Culture

A diatom strain (B-326) of *D. brightwellii* was obtained from the Korean Marine Microalgae Culture Center (KMMCC, Pukyung National University, Busan, Korea). It was cultured in f/2 medium [40] and maintained at 20 °C in a 12 h:12 h light:dark cycle with a photon flux density of approximately 65 μmol photons m^−2^ s^−1^.

### 2.2. RNA Extraction

For the RNA extraction, *D. brightwellii* cells were physically broken by homogenization using a mini-bead beater and freeze–thawing in liquid nitrogen (BioSpec Products Inc., Bartlesville, OK, USA). Total mRNA was extracted following the manufacturer’s instructions and further purified using the Mini Spin Columns from the RNeasy Mini Kit (Qiagen, Valencia, CA, USA). The extracted RNA quantity and quality were measured using a Nanoready F-1100 (Life Real, Hangzhou, China). Qualified RNA samples were used for de novo RNA transcriptome analysis and complementary DNA (cDNA) synthesis for further molecular experiments.

### 2.3. cDNA Library Construction and Transcriptome Analysis

A cDNA library of *D. brightwellii* was constructed using a TruSeq Stranded mRNA Library Prep Kit (San Diego, CA, USA) and was sequenced using an Illumina Hiseq 2500 System (2 × 150 bp). The raw sequences were analyzed using tools from the Galaxy server. In detail, a quality evaluation of the raw reads was conducted using FastQC (v0.74+galaxy1). Sequencing adapters and low-quality bases in the raw reads were trimmed using Trimmomatic (v0.39+galaxy2), and the resulting high-quality reads were assembled using Trinity (v2.15.1+galaxy1). Assembled contigs were translated into amino acid (aa) sequences through TransDecoder. LongOrfs (v5.5.0+galaxy2), and the sequences were identified through Diamond (v2.1.11+galaxy0) against the NCBI non-redundant (NR) database. By this process, identified genes and enzymes with an E value over 1.0 × 10^−15^ were cut off. Within the analyzed transcriptome of *D. brightwellii*, the sequence of *DbHsp104* was selected with keywords of ‘Hsp104’ or ‘Clp family’.

### 2.4. cDNA Synthesis, DbHsp104 Cloning, and Gene Expression Analysis

Reverse transcription was performed to synthesize cDNA using a TOPscriptTM cDNA Synthesis Kit (Enzynomics, Daejeon, Korea) with 1 μg of total RNA, random hexamers, and oligo (dT)_18_. Random hexamer was added for 5′-rapid amplification of cDNA ends (RACE) PCR and quantitative-real time PCR (qRT-PCR), and oligo (dT)_18_ was added for 3′-RACE PCR (final volume 20 μL).

The complete ORF sequence of *DbHsp104* was determined by the RACE PCR and subsequent sequencing. Specific sets of primers were designed through Primer-BLAST (https://www.ncbi.nlm.nih.gov/tools/primer-blast/, assessed on 5 May 2025). Only primer pairs with amplification efficiencies between 90% and 110% were used for analysis (Table 1). PCR amplification was performed in 20 μL of reaction mix, containing 2 μL of 10× Ex Taq buffer (TaKaRa, Japan), 2 μL of dNTP mixture (4 mM), 1 μL of each primer (10 pM), 0.2 μL of Ex Taq polymerase (2.5 U), and template cDNA. The cDNA templates were diluted by factors of 5 and 10 with nuclease-free water. All PCRs were performed using an iCycler (Bio-Rad, USA), and the conditions were as follows: pre-denaturation at 94 °C for 5 min; 35 cycles of 94 °C for 30 s, 54 °C for 30 s, 72 °C for 60 s; and extension at 72 °C for 10 min. The PCR amplicons were verified by electrophoresis on a 1.0% agarose gel, stained with Midori Green Advance (Nippon Genetics, Japan), and observed under ultraviolet light. Single bands were purified using the DNA Gel Extraction S&V Kit (Bionics, Republic of Korea) and cloned using the pMD20-T vector (Takara). The vectors were transformed into competent cells, and the recombinant plasmid sequence was determined by Sanger sequencing.

### 2.5. DbHsp104 Characterizations and Phylogeny

The amino acid sequence of Hsp104 was deduced by Translate (https://web.expasy.org/translate/, assessed on 5 May 2025) based on the obtained nucleotide sequences. The Compute pI/MW Tool (https://web.expasy.org/compute_pi/, assessed on 5 May 2025) was used to compute the isoelectric point (pI) and molecular weight (MW) properties from the aa sequences [41]. Prediction of N-terminal targeting peptides was conducted using TargetP—2.0 (https://services.healthtech.dtu.dk/services/TargetP-2.0/, assessed on 5 May 2025). Transmembrane alpha helices and signal peptides were predicted with DeepTMHMM—1.0 (https://services.healthtech.dtu.dk/services/DeepTMHMM-1.0/, assessed on 5 May 2025).

The conserved domains and active sites were identified in the InterPro database (http://www.ebi.ac.uk/interpro/, assessed on 5 May 2025). The three-dimensional (3D) structure of DbHsp104 was predicted with AlphaFold 2 (Alphafold-Colab), using MMseqs2 to generate multiple sequence alignments (MSAs) [42]. The deduced aa sequence was subjected to the Basic Local Alignment Search Tool (BLASTp) using the BLAST NR database, and similar sequences from different taxa (bacteria, cyanobacteria, diatom, dinoflagellate, fungi, green algae, and plants) were obtained (E-value < 1.0 × 10^−5^). The sequences were aligned properly by MAFFT, and their phylogenetic analysis was performed using MEGA 11 maximum likelihood (ML) phylogeny using the LG + G model with 1000 bootstrap replicates [43].

### 2.6. Thermal and Toxicant Treatments

Cells of *D. brightwellii* were cultured until an exponential phase (initial cell density 5.0 × 10^5^ cells mL^−1^) was reached, and the cultures were used for stress treatment experiments. In the present study, temperature conditions, two metals [copper (CuSO_4_ and CuCl_2_), nickel (NiSO_4_ and NiCl_2_)], and three (EDCs) [bisphenol A (BPA), polychlorinated biphenyl (PCB), and endosulfan (EDS)] were selected for these experiments. All the chemicals were purchased from Sigma-Aldrich (Sigma-Aldrich, St. Louis, MO, USA): CuSO_4_ (Cat. No. C1297), CuCl_2_ (222011), NiSO_4_ (227676), NiCl_2_ (339350), BPA (A133027), PCB (48701), and EDS (36676). The tested toxicants are considered contaminants frequently detected in coastal environments [44].

**Table 1:** The primers used in this study. Primers were used for 3′- and 5′-rapid amplification of complementary DNA ends (RACE), polymerase chain reaction (PCR), and quantitative real-time PCR (qRT-PCR).

Primer Name	Sequence (5′→3′)	Source
Db-Hsp104-F144	GGCACTTGTAGCAGGCGC	This study
Db-Hsp104-F736	CGCAAGTTGGCACAGAACG	
Db-Hsp104-F1168	GACAATGCTGTTAGAGAGGTG	
Db-Hsp104-F2002	GAGGAGTATGAAATCGTGGGA	
Db-Hsp104-ClpN-R3	CTCCAGGCTCACCCACTAA	
Db-HSP104-AAA-R1	TGCACCCACCATACGAAG	
DbHSP104-N-R1	GCTGCCTCATCAACCAAG	
DbHSP104-N-R2	CGGAGAATAGAAATGGTC	
Db-TUA-F	CGGTATCCAGACTGGTAACGGC	[45]
Db-TUA-R	GAGGCACATGCTTTCCGTTTC	
B25	GACTCTAGACGACATCGA	Universal primer
B26	GACTCTAGACGACATCGA(T)_18_	

For thermal shocks, *D. brightwellii* cells cultured at 20 °C (control) were placed into 25 °C and 30 °C incubators and harvested after 12, 24, and 48 h. In addition, diverse concentrations of metals and EDCs were treated for 24 h (Table 2), and the concentrations were determined based on the half-effective concentration (EC_50_) values examined in [45] (Table 3). Each sample was centrifuged at 2500× *g* at 4 °C for 3 min, and then the pellets were dissolved in 1 mL of TRIzol reagent (Invitrogen, Carlsbad, CA, USA). The mixtures were kept at room temperature for 5 min, frozen by liquid nitrogen, and stored at −80 °C until RNA extraction.

### 2.7. DbHsp104 Expression Analysis

The cDNA samples were diluted 1:5 with nuclease-free water for *DbHsp104* qRT-PCR. All the qRT-PCRs were performed with SYBR Green qPCR Master Mix (Noble Biosciences, Suwon, Republic of Korea) in a CFX96 Real-Time PCR Detection System (Bio-Rad Laboratories, Hercules, CA, USA). The concentration of each primer was 250 nM in the final reaction mixture. qRT-PCR conditions were as follows: 10 min at 95 °C, followed by 40 cycles of 30 s at 95 °C, 10 s at 55 °C, and 30 s at 72 °C. Relative gene expression levels were normalized using α-tubulin (*TUA*) as the internal control [45,46]. All the reactions were carried out in triplicate. The calculated mean value of Ct was used for evaluating the relative fold change based on the 2^∆∆CT^ method [47].

### 2.8. Statistical Analysis

Results in qRT-PCR were analyzed using one-way analysis of variance (ANOVA), followed by the Student–Newman–Keuls Multiple Comparisons Test to compare the relative mRNA expression. The SPSS statistical package was used for statistical analyses (Version 19.0; IBM Corp., New York, NY, USA). Probability (*p*) values of one-way ANOVA tests are indicated as * *p* < 0.05, ** *p* < 0.01, and *** *p* < 0.001. Results with *p* < 0.05 were considered significant.

## 3. Results

### 3.1. Sequence Identification of DbHsp104

**Figure 1:**
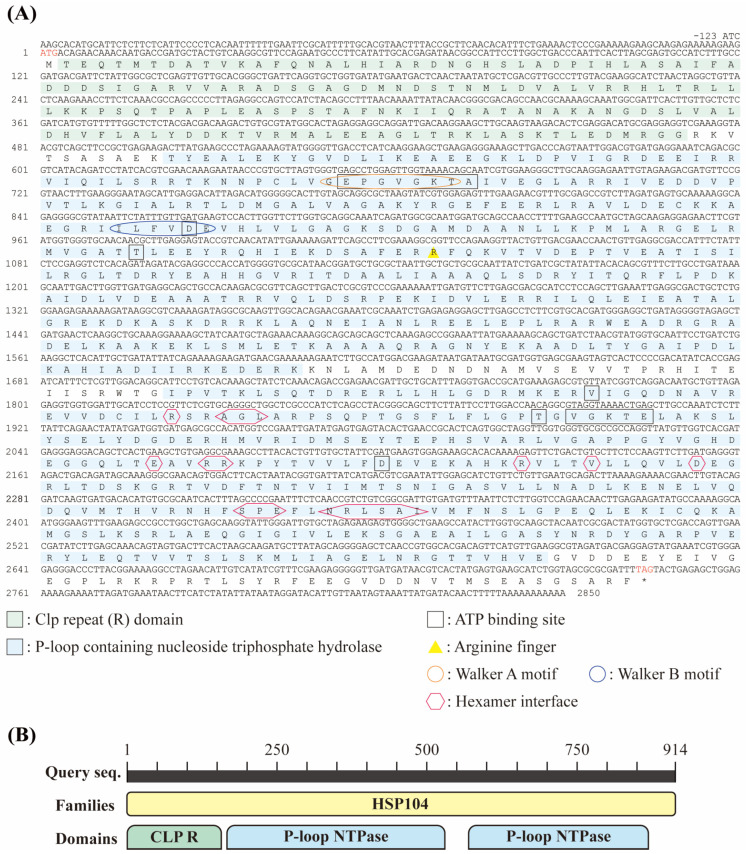
The molecular features of *DbHsp104* were determined in the marine diatom *D. brightwellii*. (**A**) Nucleotide and deduced amino acid (aa) sequences of DbHsp104. The start (ATG) and stop (TAG, *) codons are shown in red letters. Clp repeat domain (CLP R) and P-loop-containing nucleoside triphosphate hydrolase (P-loop NTPase) domain are highlighted in green and light blue, respectively. ATP-binding sites and arginine fingers are marked in a black box and a yellow triangle, respectively. Walker A and Walker B motifs are encircled in orange and blue, respectively. The hexamer interface is marked by a red octagon. (**B**) Schematic representation of the DbHsp104 showing the Hsp104 family domain (yellow), comprising the Clp repeat domain (green) and P-loop NTPase domain (light blue).

In the present study, we first determined the full cDNA sequence of *DbHsp104* from the diatom *D. brightwellii* B-326 (Figure 1A). The *DbHsp104* cDNA sequence was 2973 base pairs (bp) in length, covering an open reading frame (ORF) of 2745 bp and possessing a guanine and cytosine (GC) content of 47.7%. Untranslated regions (UTRs) were also identified, consisting of a 123 bp 5′-UTR and a 105 bp 3′-UTR (GenBank no. PV665673). The ORF started with an ATG and ended with a TAG, and the protein sequence encoded 914 aa with a MW of 101.15 kDa and a pI of 5.85. A BLASTp search of DbHsp104 showed a high identity of 79.6% (E-value, 0.0) and 77.5% (0.0) with the diatoms *Thalassiosira pseudonana* CCMP1335 (XP002288245) and *Chaetoceros tenuissimus* (GFH45977), respectively.

### 3.2. Molecular Characterization of DbHsp104

**Figure 2:**
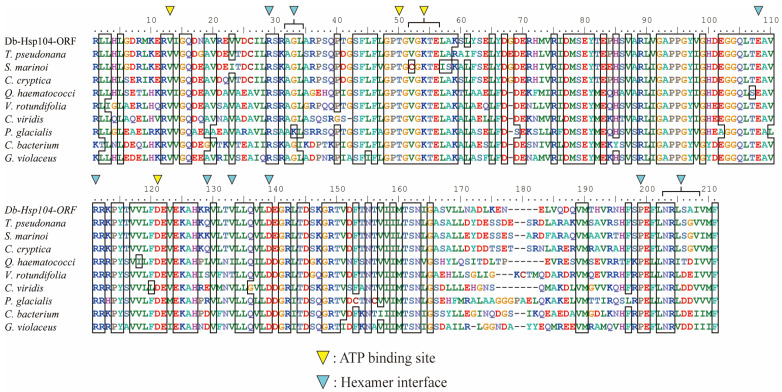
Multiple amino acid (aa) sequence alignment of DbHsp104 and the Hsp104/ClpB family. The black box represents the conserved residues among the alignments. The ATP-binding sites and hexamer interfaces are marked in yellow and blue inverted triangles, respectively. The protein sequences were taken from the GenBank database, and the details are listed in Supplementary Table S1.

TargetP-2.0 detected no signal peptide in the N-terminal of DbHsp104, and DeepTMHMM-1.0 predicted no transmembrane helices (Figure S1). The protein comprised a full-length ClpA family domain (ATP-dependent Clp protease ATP-binding subunit; COG0542) extending from residue 1 to 874 aa (Figure 1B). A Clp repeat domain (Clp R; 2–157 aa) and two P-loops that included nucleoside triphosphate hydrolase domains (P-loop NTPase; 168–535 and 571–787 aa) were identified. A multiple aa sequences of DbHsp104 and another Hsp104/ClpB were aligned (Figure 2). An ATPase AAA-type core domain (IPR003959; 192–347 aa) was located within NBD1, spanning residues 214–343 aa and containing several ATP-binding sites [EPGVGKTA^218–225^, D^289^, T^326^]. A conserved arginine finger [R^343^], Walker A motifs [GEPGVGKT^217–224^], and Walker B motifs [ILFVDE^285–290^] were also identified. Within the second loop NTPase domain, a chaperone protein ClpB/Hsp104 subfamily domain (cd19499; 580–785 aa) was found, and it contained hexamer interface residues [R^608^, AGL^611–613^, E^687^, RR^690–691^, R^708^, V^712^, D^718^, SPE^772–774^, NRLSAI^777–782^] and ATP-binding sites [V^592^, T^629^, VGKTE^631–635^, D^700^].

### 3.3. Phylogenetic Relevance and Motif Characteristic of DbHsp104

**Figure 3:**
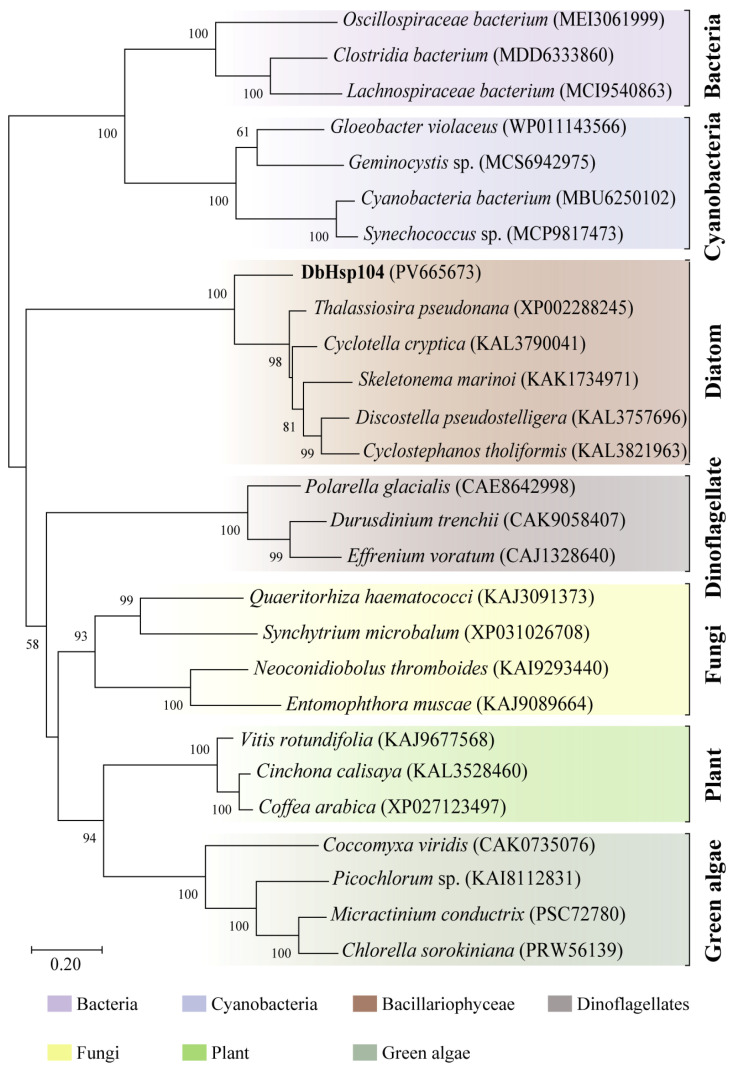
Maximum likelihood tree of DbHsp104 with other Hsp104/ClpB proteins. DbHsp104 is marked in bold. The bootstrap values under 50% were not shown. The protein sequences were taken from the GenBank database, and details are listed in Supplementary Table S2.

The ML phylogeny conducted using the LG + G model showed that DbHsp104 clustered together with the Hsp104 identified in other diatoms (Figure 3). The diatom Hsp104 formed a single clade with a maximal bootstrap value (100%), while the enzyme showed phylogenetic distance from those of other diatoms (98%). Diatom Hsp104s formed a sister clade with other eukaryotic clades (green algae, dinoflagellate, fungi, and plants; 60%). Meanwhile, eukaryotic Hsp104s and prokaryotic homologs were clearly separated, and bacteria and cyanobacterial Hsp104/Clp formed a distinct outgroup.

### 3.4. Structural Features of DbHsp104

**Figure 4:**
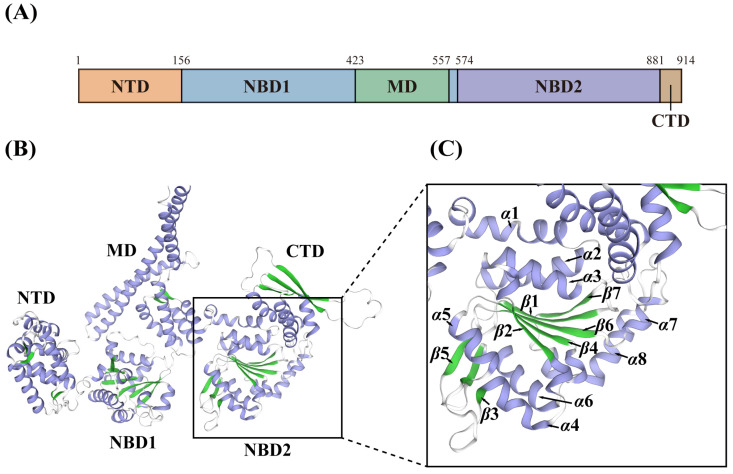
Schematic diagram of DbHsp104 domain organization (**A**). N-terminal domain (NTD), nucleotide-binding domains (NBD1 and NBD2), middle domain (MD), and C-terminal domain (CTD) are colored in orange, blue, green, blue, and brown, respectively. Three-dimensional (3D) protein structure of DbHsp104 (**B**). α-helices and β-strands are shown in purple and green, respectively (**C**).

We constructed a 3D structure model of DbHsp104 using AlphaFold2 in ColabFold, and the model was colored based on the five functional domains (Figure 4 and Figure S2). Given that DbHsp104 is a single polypeptide AAA+ chaperone that assembles into a functional homohexamer, the reliability score was determined using a predicted confidence metric (pLDDT). The top five models for DbHsp104 were provided with ranks from 1 to 5 in the order of highest reliability (Figure S3). The following graph illustrates the pLDDT value of each model. The reliability of each model was determined by measuring its performance in this particular way, and the 3D structure of the most reliable model (rank_1: model_3) was confirmed.

### 3.5. Transcriptional Changes in Dbhsp104 Under Temperature Stress

**Figure 5:**
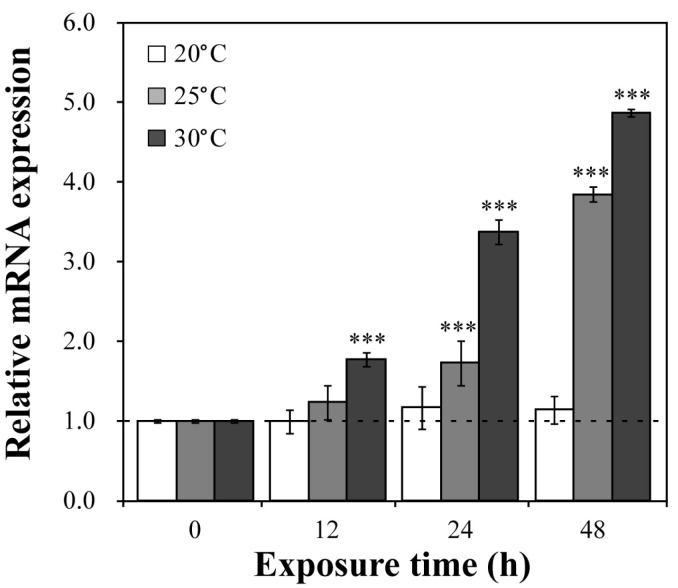
Effects of heat stress on the expression of *DbHsp104*. The gene expression levels were analyzed after exposure to three different temperatures (20, 25, and 30 °C) for four time intervals (0, 12, 24, and 48 h). Significant differences between the control and samples were determined by one-way ANOVA and are indicated as follows: *** *p* < 0.001 (n = 3). Error bars and dotted lines represent the mean standard deviation (SD) and baseline (1.0-fold) expression level of the control.

The gene expression levels of *DbHsp104* were evaluated after 0, 12, 24, and 48 h of exposure to three different temperatures (20, 25, and 30 °C) (Figure 5). Under the control condition (20 °C), no significant changes in DbHsp104 were observed, while the gene was significantly up-regulated under thermal stress. The gene expression levels at 25 °C increased 1.73-fold at 24 h and 3.85-fold at 48 h (*p* < 0.001), and they were also significantly up-regulated at 12, 24, and 48 h, with 1.77-, 3.37-, and 4.87-fold increases at 30 °C, respectively (*p* < 0.001). Taken together, the gene responses were enhanced with both temperature and exposure time.

### 3.6. Effect of Pollutants on DbHsp104 Expression

**Figure 6:**
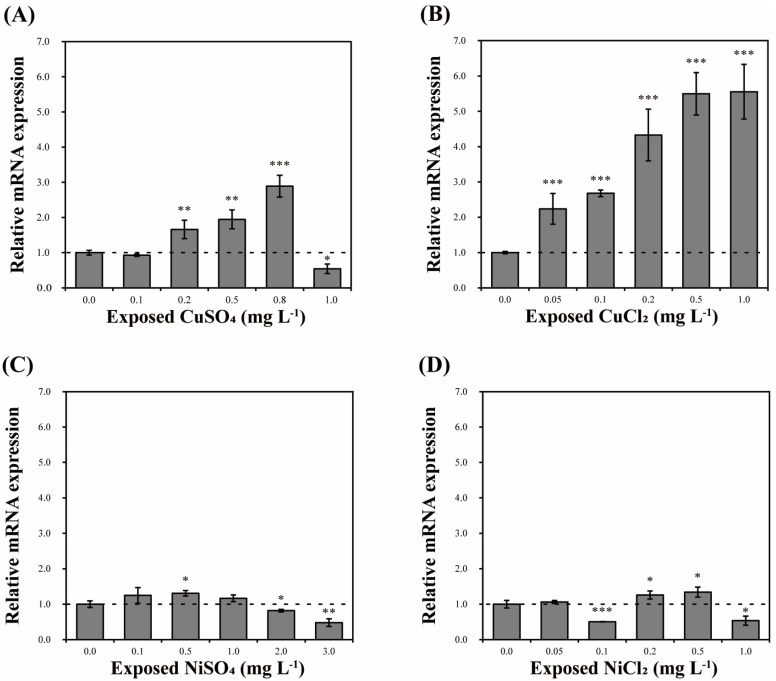
Effect of heavy metals on the expression of *DbHsp104*. *D. brightwellii* cells were exposed to various concentrations of copper sulfate (CuSO_4_, (**A**)), copper chloride (CuCl_2_, (**B**)), nickel sulfate (NiSO_4_, (**C**)), and nickel chloride (NiCl_2_, (**D**)) for 24 h. Significant differences between the control and samples were determined by one-way ANOVA and are indicated as follows: * *p* < 0.05, ** *p* < 0.01, *** *p* < 0.001 (n = 3). Error bars and dotted line represent mean standard deviation (SD) and baseline (1.0-fold) expression level of the control.

Cells of *D. brightwellii* were exposed for 24 h to five concentrations of heavy metals (CuSO_4_, CuCl_2_, NiSO_4_, and NiCl_2_) and EDCs (BPA, PCB, and EDS) to evaluate their effects on *DbHsp104* expression. The five concentrations were chosen based on the EC_50_ values of each chemical (Table 3). In the heavy metal assays, copper and nickel showed distinct patterns (Figure 6). For example, CuSO_4_ significantly up-regulated *DbHsp104* compared to the control (0.0 mg L^−1^), peaking at 0.8 mg L^−1^ (2.89-fold; *p* < 0.001); its expression rose to 0.8 mg L^−1^ and then dropped at 1.0 mg L^−1^ (0.54-fold; *p* < 0.05). CuCl_2_ showed the most significant increase in *DbHsp104* by concentration from a 2.24- to 5.55-fold increase (*p* < 0.001). In contrast, there was no notable pattern of increase or decrease in *DbHsp104* under the NiSO_4_ and NiCl_2_ treatments. The maximum increases were 1.31- and 1.34-fold at 0.5 mg L^−1^ (*p* < 0.05), and the greatest decreases were 0.48- and 0.51-fold at 3.0 mg L^−1^ of NiSO_4_ (*p* < 0.005) and 0.1 mg L^−1^ of NiCl_2_ (*p* < 0.001).

**Figure 7:**
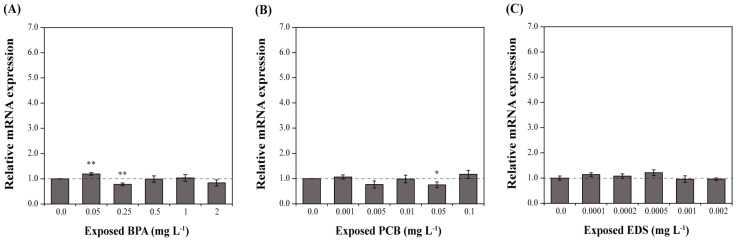
Effect of endocrine-disrupting chemicals (EDCs) on the expression of *DbHsp104*. *D. brightwellii* cells were treated with various concentrations of bisphenol A (BPA, (**A**)), polychlorinated biphenyl (PCB, (**B**)), and endosulfan (EDS, (**C**)) for 24 h. Significant differences between the control and samples were determined by one-way ANOVA and are indicated as follows: * *p* < 0.05, ** *p* < 0.01 (n = 3). Error bars and the dotted line represent the mean standard deviation (SD) and baseline (1.0-fold) expression level of the control.

Overall, EDC treatments lead to no significant changes in *DbHsp104* gene expression (Figure 7). BPA caused a slight up-regulation at 0.05 mg L^−1^ (1.19-fold; *p* < 0.05) and down-regulation at 0.25 mg L^−1^ (0.78-fold; *p* < 0.05) compared to the control (0.0 mg L^−1^). PCB reduced its expression at 0.05 mg L^−1^ (0.75-fold; *p* < 0.05), and no significant changes were observed under EDS treatments.

## 4. Discussion

### 4.1. Molecular Features of Hsp and Hsp104

Heat shock proteins (Hsps) are a family of molecular chaperones that protect cells from diverse stress conditions [48]. They play central roles in protein folding and repairing damage caused by environmental changes or pollutant exposures [21,49]. Accordingly, ecotoxicological studies have employed Hsp expression patterns as biomarkers in aquatic organisms [50,51]. Among those, the Hsp104/ClpB family shows a distinct class of ATP-dependent lyase that restores proteins from their aggregated states [52]. This function is essential for maintaining proteostasis and ensuring cellular survival under conditions that cause extensive protein denaturation.

Hsp104 has been extensively studied in the model yeast *Saccharomyces cerevisiae*, where it is vital for the induction of thermotolerance [53,54]. It was found that Hsp104 cooperates with co-chaperones Hsp40 and Hsp70 within a chaperone network to mediate protein refolding and resolubilizing aggregated proteins [54,55,56,57,58,59,60]. Structurally, Hsp104 assembles into an ATP-dependent hexameric complex that acts as a lyase and folds the aggregated polypeptide back through the central channel [61,62,63,64]. Bacterial Hsp104/ClpB proteins also show a conserved mechanism of ATP-driven protein disaggregation [34,65,66]. For example, the *Escherichia coli* ClpB is well-documented for its essential role in survival under heat shock [67,68]. In the case of microalgae, members of the Hsp104/ClpB family were identified in cyanobacteria and green algae [69,70,71]. However, studies on the structure and function of microalgal Hsp104, especially in diatoms, remain limited. Therefore, in the present study, we identified and characterized the *DbHsp104* of the marine diatom *D. brightwellii* and investigated its transcriptional responses to diverse stressors to elucidate its role in cellular stress adaptation mechanisms.

### 4.2. Structural Features of Eukaryotic DbHsp104

Bioinformatic analysis of DbHsp104 revealed that it possesses the typical molecular features of canonical Hsp104/ClpB proteins. DbHsp104 contains two highly conserved nucleotide-binding domains (NBD1 and NBD2), which include the Walker A and B motifs that function as ATP-binding and hydrolysis sites. In addition, a notable structural feature of the distal loop was found between Helix A and Helix B of the middle domain (MD). The loop enables cooperation between Hsp70 and Hsp100 and contributes to thermal resistance in vivo [30]. The distal loop sequence of DbHsp104 [ALGREKDKASKDRRK] showed a high degree of similarity (93.3%) to that of diatom *T. pseudonana* Hsp104 (GenBank accession no. XP002288245). It also shared 60% and 52.9% similarity with that of Hsp104 of the fungus *Calcarisporiella thermophila* (PDB ID: 6D00) and yeast *S. cerevisiae* (5KNE), respectively. DbHsp104 even showed about 53.3% similarity with bacterial homologs from *E. coli* (4D2U) and *Thermus thermophilus* (1QVR). These results suggest that the distal loop region is evolutionarily conserved across diverse taxa and plays a critical role in the functional activity of Hsp104/ClpB.

There was no predicted signal peptide and transmembrane domain in DbHsp104, which implies that it is a soluble non-membrane protein. In addition, it comprises five domains: NTD, NBD1, MD, NBD2, and the C-terminal domain (CTD). Structural analysis showed that MD exhibited similarity to other Hsp104/ClpB proteins. MD located between NBD1 and NBD2 serves as an important interface for interaction with the Hsp70/DnaK system, where it catalyzes substrate transfer and refolding [54,72]. Additionally, CTD is a unique feature of eukaryotic Hsp104, which is not present in bacterial ClpB orthologs [34]. Although the precise role of the CTD is not clearly defined, it is known to be related to substrate specificity and regulatory control [35]. Furthermore, phylogenetic analysis of the Hsp104/ClpB family also supports that DbHsp104 is closer to eukaryotic Hsp104 rather than bacterial ClpB. These molecular features suggest that DbHsp104 is a eukaryotic-type Hsp104 family.

### 4.3. Effect of Thermal Stress on DbHsp104 Gene Expression

Thermal stress poses a major threat to proteostasis and enzyme activity, and aquatic organisms are particularly vulnerable to such temperature fluctuations [73,74,75]. To cope with these stresses, microalgae rely on various HSPs that assist in maintaining protein homeostasis and enhancing thermotolerance [76,77,78,79]. Among those, the Hsp104/ClpB family plays a vital role in restoring protein function through resolubilization and refolding of aggregated proteins [34]. Consistently, transcriptional levels of *DbHsp104* were significantly induced by heat stress in a time- and temperature-dependent manner, showing the conserved rapid-response kinetics of the Hsp104/ClpB family. For example, *Hsp104* expression in *S. cerevisiae* yeast peaks within 1 h of exposure to approximately 39 °C [80,81]. Moreover, Yamamoto et al. [82] reported that Hsp104 transcription and protein levels increased very rapidly after heat shock, and peaked within 15 to 30 min. Similarly, *clpB* transcripts in *E. coli* and other bacteria are also rapidly up-regulated upon heat shock [83,84,85]. Taken together, our findings suggest that DbHsp104 acts as a molecular chaperone that rapidly responds to thermal stress, participates in maintaining protein homeostasis, and confers thermotolerance.

As mentioned above, Hsp104 cooperates with Hsp40/70 to mitigate proteotoxic damage and sustain cellular viability under thermal stress [54,72,86]. In our previous study, expression levels of *Hsp70* and *Hsp90* were significantly up-regulated under heat stress and peaked at 12 h at 25 °C and 48 h at 30 °C in *D. brightwellii* [87]. The results indicate a high correlation of Hsp70 and Hsp104 in their ability to repair damaged proteins caused by heat stress. These are also supported by a number of studies on Hsp responses under thermal stress in microalgae. For example, in the diatom *T. pseudonana*, both Hsp70/90 showed significant increases at 25 °C and 30 °C [77]. Similarly, *Hsp40* (*StHsp40*) was significantly up-regulated in response to heat stress in dinoflagellate *Scrippsiella trochoidea* [88]. Indeed, Santos et al. [71] reported that the mRNA levels of *Hsp70* and *Hsp100* were similarly induced in green algae *Tetraselmis suecica* under combined heat and salinity stress, which is expected as a coordinated response for thermotolerance. At the same time, Sathasivam & Ki [78] reported that qRT-PCR analysis of *Hsp70* and *Hsp100* from *T. suecica* revealed no significant changes in their expression under thermal stress. Taken together, *Hsp70* and *Hsp100* appear to regulate their expression in response to heat stress, with expression levels, peak timing, and duration varying depending on the species and strains. Across strains, their expression patterns often show similarities, which suggest coordinated regulation among Hsps.

### 4.4. Effects of Acute Toxicity on Hsp104 Transcription

A number of studies have reported that Hsps play an essential role in response to diverse environmental and ecotoxicological stresses to protect the cells and regulate the oxidative stress in microorganisms [89,90]. There are many candidates for environmental pollutants, such as heavy metals and persistent organic pollutants (POPs), including EDCs, herbicides, and antibiotics. Among those, the release of heavy metals and EDCs into the aquatic environment has been recognized as a major concern due to their long-term persistence and bioaccumulation potential [91,92]. Numerous studies have reported that such pollutants can induce oxidative stress, DNA damage, and endocrine disruption in various aquatic organisms [93,94,95]. These environmental stressors can therefore cause severe physiological and molecular disturbances, ultimately resulting in irreversible damage to aquatic life.

Copper is an essential trace metal for cell growth and is involved in respiratory electron transfer, cell wall formation, cellular defense, and diverse metabolisms in photosynthetic organisms [96]. However, it is known to cause toxicity at even low concentrations in plants and algae. In detail, excess copper inhibits growth and causes disorders in photosynthesis (chlorophyll reduction, damage in the mineral system, etc.) in its early stage [97,98]. Numerous studies of stress induced by copper also showed common phenomena of growth, chlorophyll, and photosynthesis reduction and reactive oxygen species (ROS) accumulation induced by cellular damage; green algae *Closterium ehrenbergii* [99,100,101], dinoflagellate *Cochlodinium polykrikoides* [102,103], *Prorocentrum minimum* [104,105,106], and *Palatinus apiculatus* [107], and diatoms *D. brightwellii* [45], *T. pseudonana* [77], *Nitzschia closterium* [108], *Skeletonema costatum* [109], and *Navicula* sp. [109]. The estimated EC_50_ of CuSO_4_ (0.406 mg L^−1^) was likely lowest among the heavy metal treatments, followed by NiCl_2_, CuCl_2_, and NiSO_4_ in *D. brightwellii* (Table 3). These results show that copper induces rapid and lethal toxicity in microalgae, specifically causing damage to photosynthesis-related proteins [110,111].

Such proteostasis stress induces the activation of a molecular chaperone system based on the Hsp families [112]. Consistently, *DbHsp104* was significantly induced by copper treatments, and *Hsp70*/*90* was also up-regulated by metal toxicity in *D. brightwellii* [87]. This is in good agreement with the role of Hsp104 as a chaperone that cooperates with Hsp40/70 to remodel and recover damaged proteins [54,58]. In this regard, Sathasivam & Ki [78] reported that expression of *Hsp70*/*100* was significantly induced in green algae *T. suecica*, while *Hsp20* decreased. Moreover, mRNA levels of *Hsp70* were up-regulated in dinoflagellate *P. minimum* and green algae *C. ehrenbergii* under copper exposures [46,79]. Notably, *Hsp70* shows strong induction in acute damage, involved in the early stages of protein repair [113]. Collectively, the results suggest that DbHsp104 responds rapidly to acute stress and is likely to cooperate with Hsp70 to manage initial proteostasis recovery.

### 4.5. Minor Effects of EDCs on Hsp104 System

EDCs are substances that interfere with the transmission of hormonal signals [114]. Surprisingly, a number of studies have reported that EDCs can inhibit the growth of non-target microorganisms [114,115,116]. Although the mechanisms of reaction and sensitivity vary depending on the compounds, the three tested EDCs have been shown to be able to delay growth by interfering with the cell cycle [115]. In addition, BPA [114] and EDS [116] are reported to induce oxidative stress and membrane/permeability disruption in microalgae. Similarly, Wang et al. [115] reported that PCBs did not increase the expression level of *Hsp90* in the dinoflagellate *P. minimum*, while they decreased the expression of *Hsp90* in the dinoflagellate *C. polykrikoides* [117]. In addition, neither the *Hsp90* of green algae *C. ehrenbergii* [79] nor the *hsp70*/*83* of fruit fly *Drosophila melanogaster* [118] reacted to EDS. In line with these, EDCs yielded higher EC_50_ values compared to the heavy metals and did not show an acute toxic signature or fatal mortality. Consistently, *DbHsp104* showed no significant transcriptional induction under exposure to any of the three EDCs. These results support the finding that EDCs induce subacute stress related to cell division, leading to damage that is less dependent on Hsp104.

### 4.6. DbHsp104 as a Potential Biomarker

The distinct expression profile of *DbHsp104* highlights it as a potential biomarker for environmental stresses, especially acute toxicity. It showed a rapid and dose-dependent transcriptional response to key stressors, heat and copper, both common and potent water pollutants [91,119]. Unlike common biomarkers that respond to multiple stress factors, the observed response of *DbHsp104* suggests it may have potential in more specific diagnostic applications [120]. Monitoring *DbHsp104* and *Hsp104/clpB* from other photosynthetic organisms thus provides a sensitive and early warning of water contamination, enabling more rapid monitoring and active management [120,121]. Future studies would benefit from extending more diverse Hsp104 candidates to test their responses to diverse metals (e.g., cadmium, lead, zinc) and contaminants to establish their applications.

## 5. Conclusions

The present study extends the previous study by Lee et al. [45] regarding the high sensitivity of *Hsp* in diatom *D. brightwellii* to various toxic chemicals. To our knowledge, this is the first report identifying the molecular features of DbHsp104 and evaluating its transcriptional responses in diatoms. Phylogeny and domain organization showed the distinctive features of the eukaryotic Hsp104/ClpB family. Transcriptional responses exhibited significant induction under heat stress and copper exposure. There was no change in response to nickel or the tested EDCs. Collectively, the results indicate a selective and distinct responsiveness to acute thermal and copper stress, suggesting that DbHsp104 may contribute to the rapid restoration of proteostasis. These findings suggest that *Hsp104* may be a useful molecular biomarker for environmental monitoring using diatoms. Future work should integrate an analysis of Hsp104 at the protein level.

## Figures and Tables

**Table 2:** The chemicals used in the exposure experiments and their treatment concentrations. The tested substances included four heavy metals [copper sulfate (CuSO_4_), copper chloride (CuCl_2_), nickel sulfate (NiSO_4_), and nickel chloride (NiCl_2_)] and three endocrine-disrupting chemicals [bisphenol A (BPA), polychlorinated biphenyl (PCB), and endosulfan (EDS)].

Chemicals	Treatments (mg L^−1^)					
CuSO_4_	0.0	0.1	0.2	0.5	0.8	1.0
CuCl_2_	0.0	0.05	0.1	0.2	0.5	1.0
NiSO_4_	0.0	0.1	0.5	1.0	2.0	3.0
NiCl_2_	0.0	0.05	0.1	0.2	0.5	1.0
BPA	0.0	0.05	0.25	0.5	1.0	2.0
PCB	0.0	0.001	0.005	0.01	0.05	0.1
EDS	0.0	0.0001	0.0002	0.0005	0.001	0.002

**Table 3:** The half-maximal effective concentrations (EC_50_) of four heavy metals [copper sulfate (CuSO_4_), copper chloride (CuCl_2_), nickel sulfate (NiSO_4_), and nickel chloride (NiCl_2_)] and three endocrine-disrupting chemicals [bisphenol A (BPA), polychlorinated biphenyl (PCB), and endosulfan (EDS)] were determined from the marine diatom *D. brightwellii*.

Chemicals	EC_50_ (mg L^−1^)	Reference
CuSO_4_	0.406	[45]
CuCl_2_	1.455	
NiSO_4_	3.468	
NiCl_2_	0.720	
BPA	0.039	
PCB	0.002	
EDS	0.001	

## Data Availability

The data presented in this study are available on request from the corresponding author.

## Supplementary Materials

The following supporting information can be downloaded at https://www.mdpi.com/article/10.3390/genes16121408/s1. Table S1: Species and accession numbers of Hsp104/ClpB amino acid sequences used for the alignments; Table S2: Taxon, species, and accession numbers of Hsp104/ClpB amino acid sequences used for phylogenetic tree analysis; Figure S1: (A) TargetP-2.0 prediction for the signal peptide and (B) TMHHM-2.0 prediction for transmembrane helices of the DbHsp104 identified from the marine diatom *D. brightwellii*; Figure S2: (A) Domain organization and (B) three-dimensional structure (3D) of the DbHsp104 identified from the marine diatom Ditylum brightwellii. Three-dimensional structure of DbHsp104 color-coded by the domains; N-terminal domain (NTD, orange), nucleotide-binding domain 1 (NBD1, blue), middle domain (MD, green), NBD2 (purple), and C-terminal domain (CTD, brown); Figure S3: Residue confidence profiles for the five top-ranked AlphaFold2 models of DbHsp104. The plot shows predicted IDDT (pLDDT) values for residues 1–915 aa on the *x*-axis and scores from 0 to 100 on the *y*-axis (≥90, very high; 70–90, high; <70, moderate–low confidence). Curves for rank 1–5 (blue, orange, green, red, purple) overlap across the sequence; Figure S4: Conserved residues mapped onto the three-dimensional (3D) structure of DbHsp104. (A) Overview of residues of nucleotide-binding domain 1 (NBD1). (B) Walker A motif (orange), Walker B motif (blue), and conserved arginine finger (yellow). (C) Overview of residues of NBD2. (D) Hexamer interface (red). (E) Overview of ATP-binding sites. (F) Specific ATP-binding site (black).

## Abbreviations

The following abbreviations are used in this manuscript:

HSP	heat shock protein
CuSO_4_	copper sulfate
CuCl_2_	copper chloride
NiSO_4_	nickel sulfate
BPA	bisphenol A
PCB	polychlorinated biphenyl
EDS	endosulfan
EDCs	endocrine-disrupting chemicals
P-loop NTPase	P-loop containing nucleoside triphosphate hydrolase
NTD	N-terminal domain
NBD	nucleotide-binding domains
CTD	C-terminal domain
CLP	R Clp repeat domain
ML	Maximum likelihood
ANOVA	One-way analysis of variance
