# Supplementary material for: Heat Shock Protein 104 (Hsp104) in the Marine Diatom Ditylum brightwellii: Identification and Transcriptional Responses to Environmental Stress
Source: Genes (Basel). 2025 Nov 26;16(12):1408. doi: 10.3390/genes16121408 (PMC12732369; doi:10.3390/genes16121408)
Supplement: Supplementary file 1 [file genes-16-01408-s001.zip › Supplementary Files/2025-11-DbHsp_Suppl_Figs.pptx]

## Slide 1
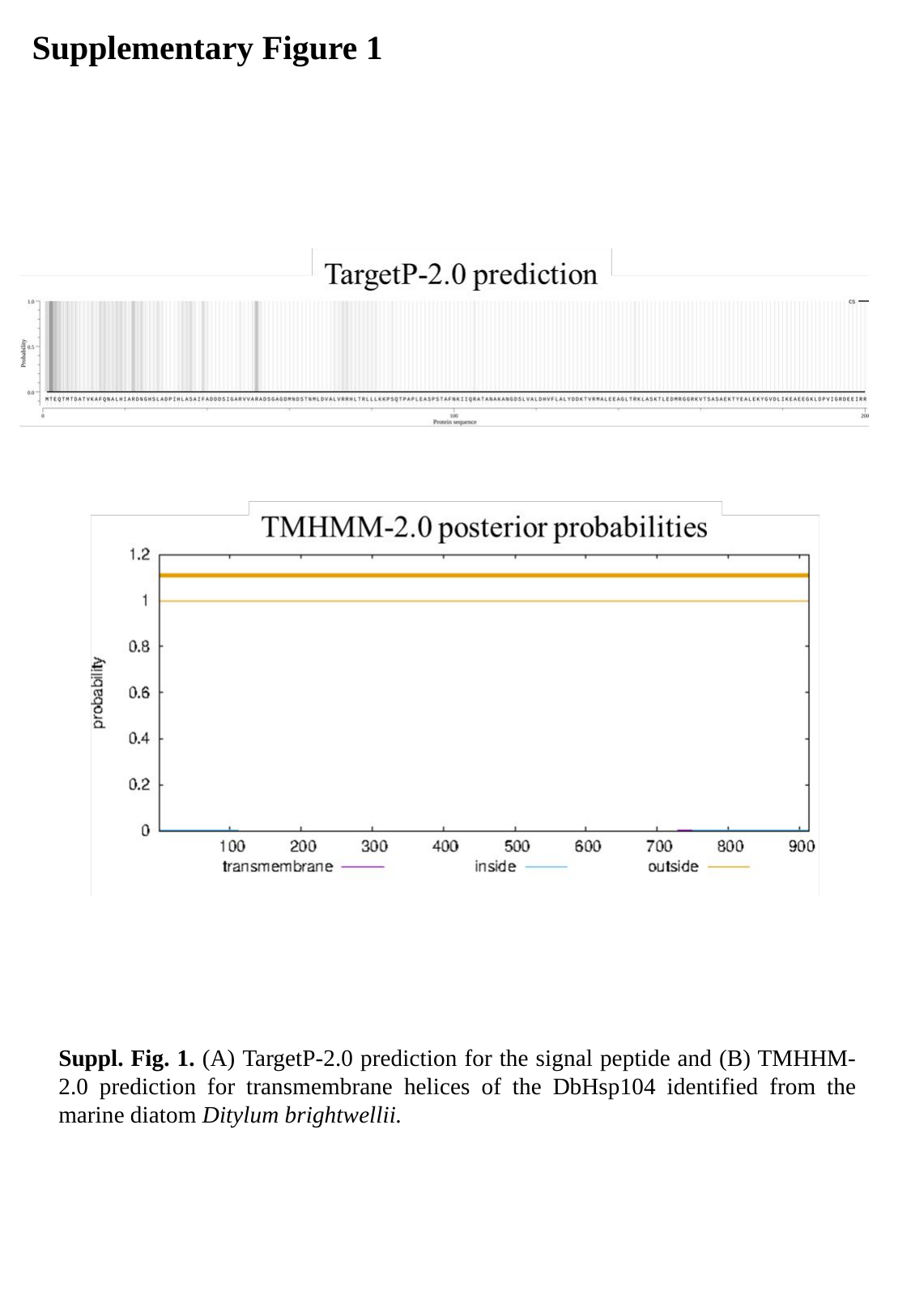

Supplementary Figure 1
Suppl. Fig. 1. (A) TargetP-2.0 prediction for the signal peptide and (B) TMHHM-2.0 prediction for transmembrane helices of the DbHsp104 identified from the marine diatom Ditylum brightwellii.

## Slide 2
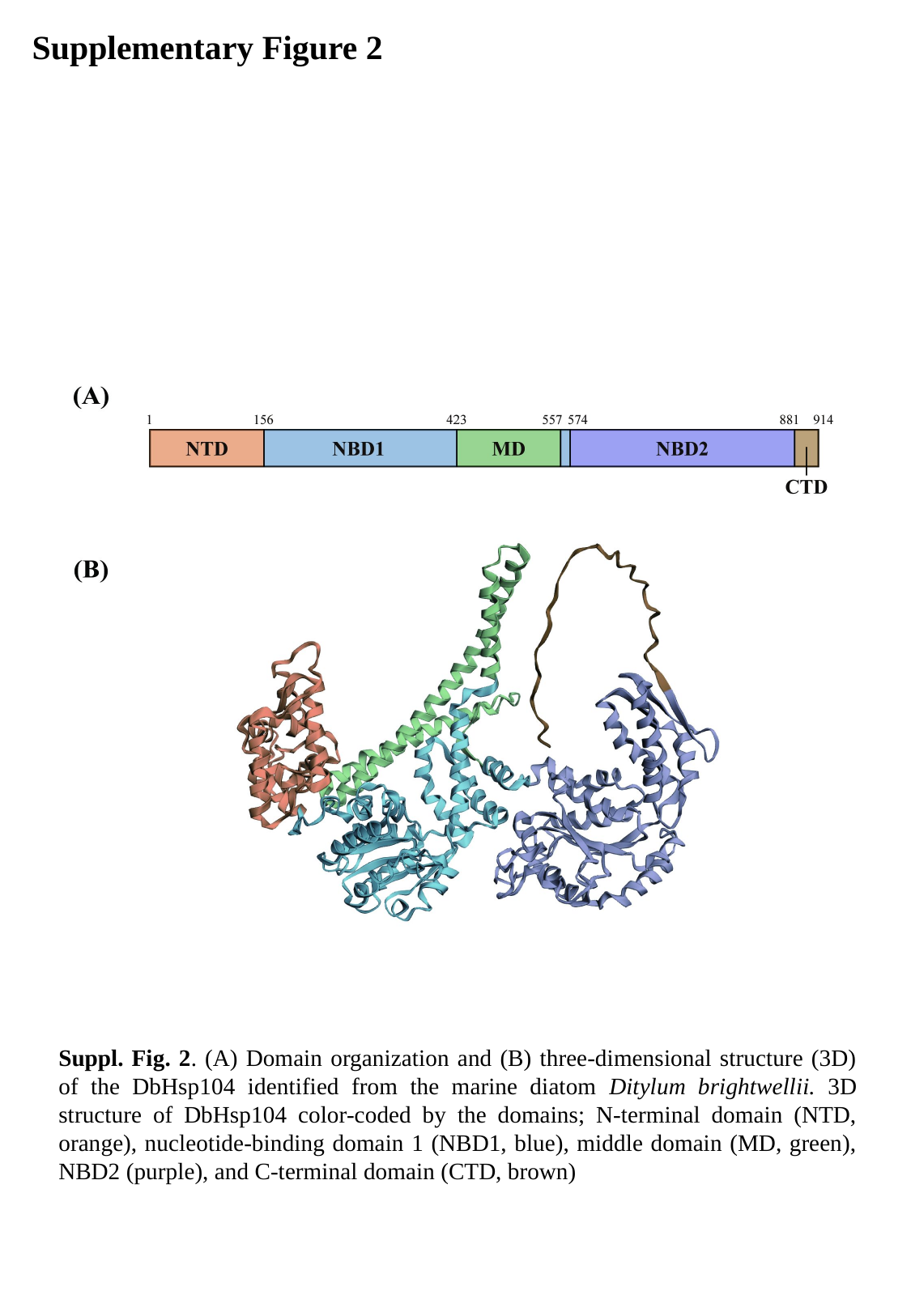

Supplementary Figure 2
Suppl. Fig. 2. (A) Domain organization and (B) three-dimensional structure (3D) of the DbHsp104 identified from the marine diatom Ditylum brightwellii. 3D structure of DbHsp104 color-coded by the domains; N-terminal domain (NTD, orange), nucleotide-binding domain 1 (NBD1, blue), middle domain (MD, green), NBD2 (purple), and C-terminal domain (CTD, brown)

## Slide 3
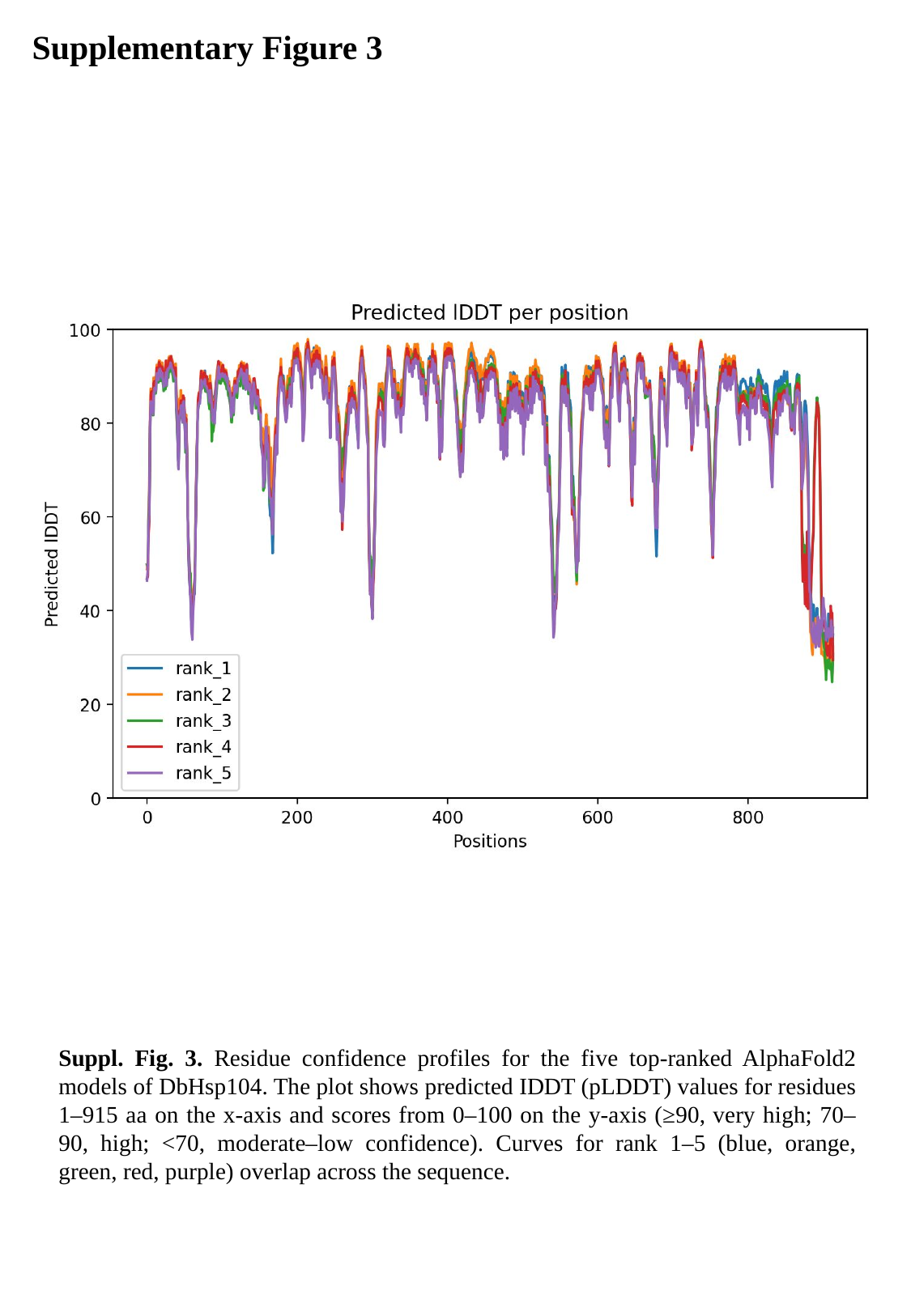

Supplementary Figure 3
Suppl. Fig. 3. Residue confidence profiles for the five top-ranked AlphaFold2 models of DbHsp104. The plot shows predicted IDDT (pLDDT) values for residues 1–915 aa on the x-axis and scores from 0–100 on the y-axis (≥90, very high; 70–90, high; <70, moderate–low confidence). Curves for rank 1–5 (blue, orange, green, red, purple) overlap across the sequence.

## Slide 4
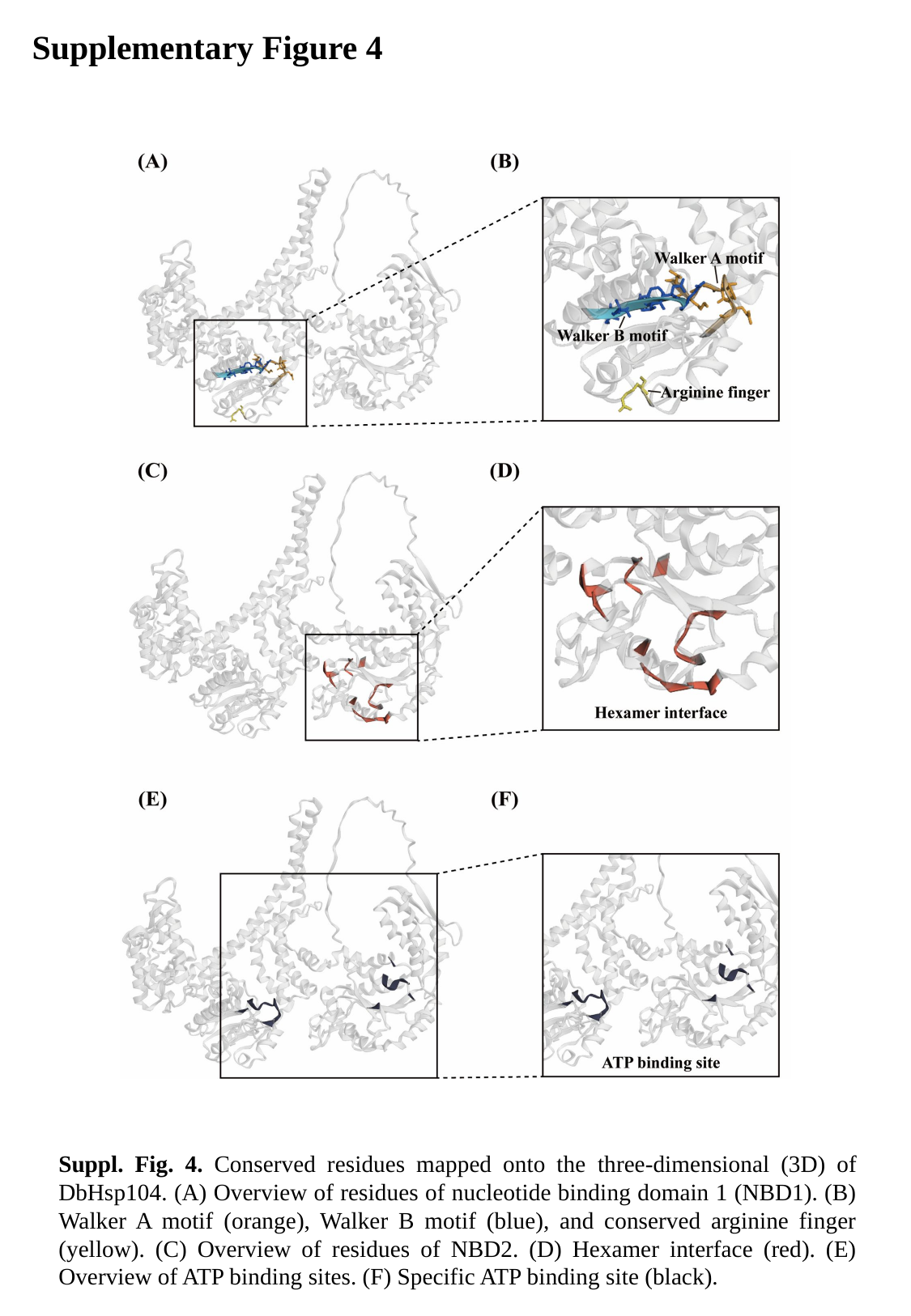

Supplementary Figure 4
Suppl. Fig. 4. Conserved residues mapped onto the three-dimensional (3D) of DbHsp104. (A) Overview of residues of nucleotide binding domain 1 (NBD1). (B) Walker A motif (orange), Walker B motif (blue), and conserved arginine finger (yellow). (C) Overview of residues of NBD2. (D) Hexamer interface (red). (E) Overview of ATP binding sites. (F) Specific ATP binding site (black).
